# The dynamics of gene expression changes in a mouse model of oral tumorigenesis may help refine prevention and treatment strategies in patients with oral cancer

**DOI:** 10.18632/oncotarget.8321

**Published:** 2016-03-24

**Authors:** Jean-Philippe Foy, Antonin Tortereau, Carlos Caulin, Vincent Le Texier, Emilie Lavergne, Emilie Thomas, Sylvie Chabaud, David Perol, Joël Lachuer, Wenhua Lang, Waun Ki Hong, Patrick Goudot, Scott M Lippman, Chloé Bertolus, Pierre Saintigny

**Affiliations:** ^1^ INSERM U1052, Cancer Research Center of Lyon, Lyon, France; ^2^ CNRS UMR 5286, Cancer Research Center of Lyon, Lyon, France; ^3^ Department of Oral and Maxillofacial Surgery, University of Pierre Marie Curie-Paris 6, Pitié-Salpêtrière Hospital, Paris, France; ^4^ Université de Lyon, VetAgro Sup, UPSP 2011-03-101, ICE, Marcy-l'Étoile, France; ^5^ Head and Neck Surgery at The University of Texas MD Anderson Cancer Center, Houston, TX, USA; ^6^ Department of Bioinformatics, Centre Léon Bérard, Lyon, France; ^7^ Department of Biostatistics, Centre Léon Bérard, Lyon, France; ^8^ Université Lyon 1, Université de Lyon, Lyon, France; ^9^ ProfileXpert, SFR-Est, CNRS UMR-S3453, INSERM US7, Lyon, France; ^10^ Thoracic/Head and Neck Medical Oncology at The University of Texas MD Anderson Cancer Center, Houston, TX, USA; ^11^ Division of Cancer Medicine at The University of Texas MD Anderson Cancer Center, Houston, TX, USA; ^12^ UC San Diego Moores Cancer Center, La Jolla, CA, USA; ^13^ Departments of Medicine and Translational Research and Innovation, Centre Leon Berard, Lyon, France; ^14^ Centre Léon Bérard, Lyon, France

**Keywords:** oral preneoplasia, tumorigenesis, prevention, oral cancer, genome-wide expression profiles

## Abstract

A better understanding of the dynamics of molecular changes occurring during the early stages of oral tumorigenesis may help refine prevention and treatment strategies. We generated genome-wide expression profiles of microdissected normal mucosa, hyperplasia, dysplasia and tumors derived from the 4-NQO mouse model of oral tumorigenesis. Genes differentially expressed between tumor and normal mucosa defined the “tumor gene set” (TGS), including 4 non-overlapping gene subsets that characterize the dynamics of gene expression changes through different stages of disease progression. The majority of gene expression changes occurred early or progressively. The relevance of these mouse gene sets to human disease was tested in multiple datasets including the TCGA and the Genomics of Drug Sensitivity in Cancer project. The TGS was able to discriminate oral squamous cell carcinoma (OSCC) from normal oral mucosa in 3 independent datasets. The OSCC samples enriched in the mouse TGS displayed high frequency of *CASP8* mutations, 11q13.3 amplifications and low frequency of *PIK3CA* mutations. Early changes observed in the 4-NQO model were associated with a trend toward a shorter oral cancer-free survival in patients with oral preneoplasia that was not seen in multivariate analysis. Progressive changes observed in the 4-NQO model were associated with an increased sensitivity to 4 different MEK inhibitors in a panel of 51 squamous cell carcinoma cell lines of the aerodigestive tract. In conclusion, the dynamics of molecular changes in the 4-NQO model reveal that MEK inhibition may be relevant to prevention and treatment of a specific molecularly-defined subgroup of OSCC.

## INTRODUCTION

Personalization of prevention strategies may allow the decrease of morbidity and mortality of head and neck squamous cell carcinoma (HNSCC), the second most common smoking-related cancer after lung cancer [1]. Previous reports on head and neck premalignancy and chemoprevention have focused on oral squamous cell carcinoma (OSCC), the most common anatomical site of HNSCC. Although prospective evaluations of chemopreventive agents have not yet resulted in the development of an intervention that can be considered as standard of care, they allowed the identification of new biomarkers of OSCC risk in patients with oral premalignant lesions (OPL), the most validated one being loss of heterozygosities (LOH) at various sites [2–4].

Despite those efforts, the natural history of OPL remains poorly understood, as they may persist for years, regress spontaneously or after tobacco or alcohol cessation, or evolve into invasive SCC in about 20% cases over periods of up to 30 years [5–8]. A comprehensive characterization of the molecular changes occurring in OPL may improve our understanding of their natural history [7–10]. We have previously shown that gene expression profiles of OPL may improve the prediction of oral cancer [11]. However, the study was limited to patients treated with retinoids and to the analysis of a single baseline biopsy, which did not allow us to appreciate the dynamics of gene expression changes over time.

In order to address these limitations and to identify key molecular changes leading to the development of cancer, we sought to characterize the dynamics of gene expression changes in the 4-nitroquinoline 1-oxide (4-NQO) mouse model of human oral carcinogenesis that leads to the development of malignant SCC preceded by preneoplastic histological changes such as hyperplasia and dysplasia [12–16]. Progression of premalignant pathologic stages has been well described in this model [14], showing hyperplasia at first weeks after 4-NQO administration and then an increasing percentage of dysplasia and tumors, reproducing the multi-step tumor process observed in humans. This model represents a relevant model of human SCC as it is associated with genetic alterations similar to those observed with smoking exposure in HNSCC (*i.e.* EGFR overexpression) [13, 16]. However, the 4-NQO mouse model has not been extensively characterized at the molecular level.

In the present study, we characterized genome-wide expression changes in the 4-NQO mouse model and identified gene sets specific of sequential histological changes and demonstrated the relevance of those changes in human OSCC. An integrative analysis of the TCGA data showed that the 4-NQO model may represent a specific molecularly-defined subgroup of OSCC, and may be associated with patterns of drug sensitivity in human *in vitro* models of aerodigestive SCC. The study of the dynamics of gene expression changes during oral tumorigenesis may help refine prevention and treatment strategies.

## RESULTS

### Dynamics of gene expression changes during oral tumorigenesis in the 4-NQO mouse model

**Figure 1:**
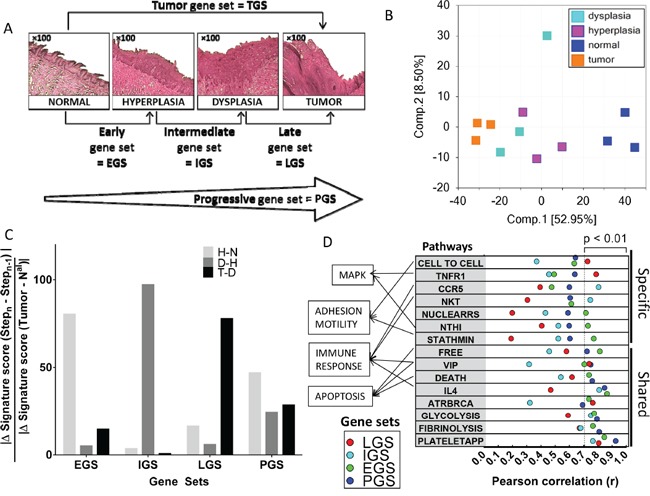
Dynamic gene expression changes during oral tumorigenesis in the 4-NQO mouse model and their association with canonical pathways **A.** Histologic changes during oral carcinogenesis in the mouse model and corresponding gene set for each step of this tumor process: normal, hyperplastic, dysplastic and tumor mucosa. **B.** Principal component analysis including the 12 samples and using whole-genome. **C.** An enrichment rate was computed for early (EGS), intermediate (IGS), late (LGS) and progressive (PGS) gene sets at each step of oral tumorigenesis, i.e. between normal and hyperplasia (H-N), hyperplasia and dysplasia (D-H), and dysplasia and tumor (T-D) (see Material and Methods section). **D.** Enrichment scores for EGS, IGS, LGS and PGS gene subsets were correlated with enrichment scores for 208 BIOCARTA pathways (see Material and Methods section); pathways with the strongest correlation at each step of oral tumorigenesis are shown and ranked according to the Pearson's r (F).

Nine mice treated by the 4 NQO as described elsewhere [14] and three control mice (non-treated) were selected for transcriptomic analysis. Total RNA extraction was performed from microdissected epithelial cells of tongue mucosa, including 3 normal mucosa from control mice (week 12, 20, and 24), 3 hyperplastic lesions (1 at week 16 and 2 at week 20), 3 dysplastic lesions (2 at week16, 1 at week 24) and 3 tumor samples (at week 16, 20 and 24) from the 4-NQO treated mice (Figure 1A). Median RIN was 7.55 (range 4.6-9.2) with no significant difference between normal, hyperplastic, dysplastic and tumor samples. RNA concentration ranged between 0.5 and 5.7 ng/μL, with a median of 1.2 ng/μL. All of the 12 samples passed the quality controls, were amplified and arrays were hybridized on Mouse Gene ST 2.0 Arrays. The technical information is detailed in Supplementary Table 1.

A principal component analysis (PCA) of the 12 samples using whole-genome expression profiles demonstrated strong patterns of normal mucosa, hyperplastic, dysplastic and tumors lesions as shown in Figure 1B. We then identified several sets of genes that were associated with histological changes (Figure 1A). Differential expression was defined by an absolute log2 fold-change (FC) >1. Each gene set was divided into an “UP” version for genes upregulated (log2 FC > 1) and a down (DN) version for genes downregulated (log2 FC <-1), respectively. Five mouse gene sets were defined and human orthologous genes were identified for each gene set, using the JAX and HUGO orthology resources [17, 18]. We first identified a set of genes differentially expressed between tumor and normal mucosa that we called the “tumor gene set” (TGS). TGS included 584 and 753 mouse genes that were up- and down-regulated genes in tumors, respectively, and that correspond to 514 and 508 human orthologous genes respectively (Supplementary Table 2). A PCA of the 12 samples using TGS demonstrated a similar pattern as the one observed using whole-genome expression profiles (Supplementary Figure 1A).

In order to capture the dynamics of gene expression changes during oral tumorigenesis, we subsequently divided the TGS set into four non-overlapping gene subsets: the “early” (EGS), the “intermediate” (IGS) and the “late” (LGS) gene subsets, which represented the changes observed at each step of oral tumorigenesis (i.e. hyperplasia vs. normal mucosa, dysplasia vs. hyperplasia and tumor vs. dysplasia respectively). Genes included in EGS, IGS and LGS were differentially expressed at a given stage of oral tumorigenesis but not differentially expressed at any previous stage. The fourth gene subset, “progressive” gene set (PGS), represented changes acquired progressively during each step, that reached an absolute log2 FC > 1 in tumor vs. normal mucosa, but did not reach this FC at any individual steps of oral tumorigenesis (Supplementary Table 3). EGS and PGS included the largest number of genes (Supplementary Table 2). Details of the genes included in each gene set are provided in Supplementary Table 4.

In order to gain insight on the biological significance of genes differentially expressed in mice tumors vs. normal mucosa, a gene set enrichment analysis (GSEA) was performed to determine whether an *a priori* defined set of genes showed statistically significant differences between tumor vs normal samples [19]. The log_2_ FC was used to successively analyze a total of 4,722 “curated gene sets” (C2) and 189 “oncogenic signatures” (C6) available from the MSigDB [19]. The most significant gene sets with a positive enrichment score among “curated gene sets” were related to proliferation and cell cycle (Supplementary Figure 1B). EGFR and VEGF pathways were the most significantly enriched gene sets with a positive score, among the 189 “oncogenic signatures” (Supplementary Figure 1C).

We then used the single sample GSEA (ssGSEA) [19, 20] to compute an enrichment score (ES) for the four subsets (EGS, IGS, LGS and PGS) in each of the 12 mouse samples. The specificity of each gene subset for a given stage of oral tumorigenesis was confirmed by an enrichment rate calculated for each gene subset at each step, using the formula: |Δ GeneSet Score (Step_n_-Step_n-1)_| / |ΔGene Set score (Tumor-Nal)|. The rates of enrichment score for ESG, IGS, and LGS were 80%, 97% and 78%, respectively (Figure 1C). In contrast, the rate of ES of the PGS was well distributed across different steps of oral tumorigenesis (24%, 29% and 47%).

In order to test the biological significance of EGS, IGS, LGS and PGS in the 12 mouse samples, we computed their enrichment score and tested their correlation with the enrichment score for 208 Biocarta pathways. We found 15 pathways correlated with at least one gene set using a *P* <0.01 (see Supplementary Tables 5 and 6) (Figure 1D). Two of the pathways involve the mitogen-activated protein kinases (MAPK) signaling pathways. The first one, “NTHI”, corresponds to an inflammatory response through the activation of NF-κB and p38/MAPK and was mostly correlated with EGS (r=0.73, *P*=0.0070). The second pathway, “TNFR1”, involves apoptosis and the JNK/MAPK pathway, and was mostly correlated with LGS (r= 0.79, *P*=0.0023). Additional pathways correlated with the gene subsets were immune-related. “CCR5” and “NKT” were mostly correlated with the IGS (r=0.81, *P*=0.0016 and r=0.75, *P*=0.0053 respectively) while “IL4” pathway was highly correlated with EGS (r=0.86, *P*=0.0003), IGS (r=0.81, *P*=0.0014) and PGS (r=0.85, *P*=0.0005). Apoptosis was represented by “DEATH” and “FREE” pathways, mostly correlated with EGS (r=0.74, *P*=0.0061 and r=0.81, *P*=0.0013 respectively) and PGS (r=0.76, *P*=0.0041 and r=0.72, *P*=0.0078 respectively).

### The 4-NQO model derived TGS discriminates OSCC from normal mucosa and identifies OSCC with specific genomic alterations

**Figure 2:**
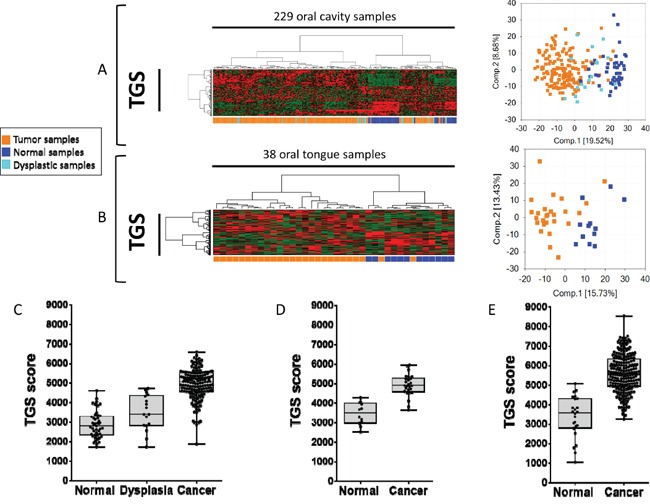
The tumor gene set (TGS) identified in the 4-NQO model of oral tumorigenesis is relevant to human disease and discriminates oral squamous cell carcinoma (OSCC) from normal mucosa samples Hierarchical clustering and principal component analysis (PCA) using the TGS in: a set of 45 normal oral mucosa samples, 17 oral dysplasia, 167 OSCC (GSE30784) [22]. **A.** and a set of 12 normal tongue samples and 26 Oral Tongue SCC (OTSCC) (GSE9844) [21] **B.** Enrichment scores (ES) were computed for the TGS in GSE30784 **C.**, GSE9844 (D), and in the TCGA set of 221 OSCC and 24 normal oral mucosa samples (E). Statistical significance was given by a Mann Whitney test **D and E.** or a Kruskal-Wallis test (C). We observed a significant difference (*P*<0.0001) between ES of normal, dysplastic and tumor samples in all datasets.

In order to test the relevance of the 4-NQO model in human disease, we first determined whether the TGS was able to discriminate cancer vs. normal tissues in three datasets downloaded from public repositories, using array-based or RNAseq expression profiles (Supplementary Table 7). A PCA and unsupervised hierarchical cluster analysis was performed using the TGS in two independent human datasets: GSE9844 [21] and GSE30784 [22]. Two main clusters were observed in GSE30784 (Figure 2A): the first one included no normal samples (0/45), 89% (151/169) of tumor samples and 59% (10/17) of dysplastic samples, whereas the second one included all normal samples (45/45), 11% (18/169) of tumor samples and 41% (7/17) of dysplastic samples (Fisher's exact test *P* <0.0001). Similar results were obtained in GSE9844 (Figure 2B) with one cluster including 92% (24/26) of OSCC of the tongue and no normal mucosa, while the other one included 8% (2/26) of OSCC of the tongue and all the normal samples (Fisher's exact test *P* <0.0001). We then computed the enrichment score (ES) of each sample for the TGS using ssGSEA and consistently found higher ES in cancer vs. normal mucosa samples in GSE30784 (Figure 2C) and GSE9844 (Figure 2D). Interestingly, dysplasia had intermediate scores, some of them overlapping with tumors, while others overlapped with normal mucosa (Figure 2C). These results were validated in a TCGA set [23] of 221 OSCC and 24 normal mucosa samples (Figure 2E). 4-NQO is a DNA adduct-forming agent that serves as a surrogate of tobacco exposure. Interestingly, we evidenced that TGS ES scores were higher in smokers and drinkers (SD) compared to never-smokers and never drinkers (NSND) (*P*=0.0400) (Supplementary Figure 2).

**Figure 3:**
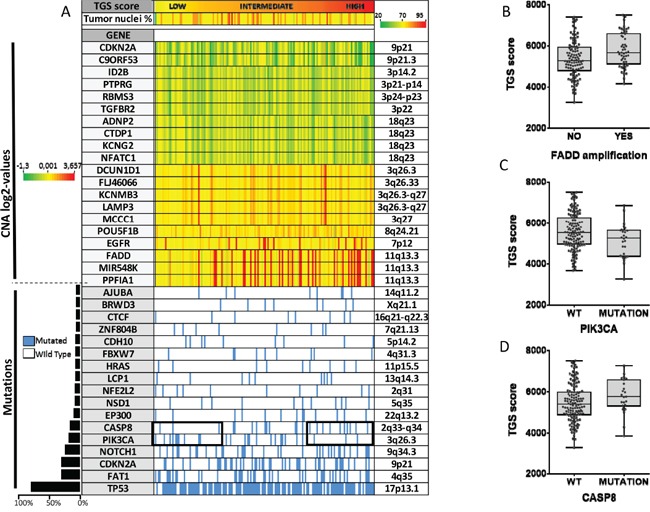
An integrated analysis reveals that human oral squamous carcinomas (OSCC) enriched for the mouse tumor gene set (TGS) harbor specific genomic alterations Copy number (CN) alterations, somatic mutation status and RNA sequencing data were analyzed for 152 patients with OSCC from the TCGA. Patients are represented by columns while specific genes are represented by rows. Tumors are ranked by the TGS enrichment score. The genes most frequently deleted (n=10) or amplified (n=10) in HNSCC, or that were found to be mutated in at least 5% of HNSCC (n=17) are shown (see Material and Methods section). Percent of malignant cells is shown below the TGS enrichment score **A.** TGS enrichment scores according to FADD amplification **B.** *CASP8* **C.** and *PIK3CA* **D.** mutation status are shown. Statistical significance was given by a Student's test (B) and a Mann-Whitney test (C, D). We found a higher TGS score in OSCC harboring FADD amplification (*P*=0.0039), CASP8 mutation (*P*=0.0595), and wild type PIK3CA (*P*=0.0319).

We then tested whether the 4-NQO model was representing a subgroup of OSCC. For this purpose, we performed an analysis integrating gene expression, mutation and copy number (CN) data available for 152 patients with OSCC in the TCGA. Although TGS ES was not associated with the percent of malignant cells, OSCC tumors were ranked according to the TGS ES (Figure 3). Tumors enriched for TGS were associated with copy number gains in 11q13.3, a region that includes in particular *FADD*, *MIR548K* and *PPFIA1*3. A significant correlation was found between the TGS ES and *FADD* absolute CN (r=0.27, *P*=0.0007) (data not shown). Using an absolute CN > 3 to define an amplification, we showed that OSCC harboring *FADD* amplification had a significant higher TGS score (*P*=0.0039). Furthermore, higher TGS ES were observed in OSCC from patients with *CASP8* mutations (*P*=0.0595) and WT *PIK3CA* (*P*=0.0319) (Figure 3B, 3C, 3D).

### The 4-NQO derived gene sets and their association with drug sensitivity patterns *in vitro*

**Figure 4:**
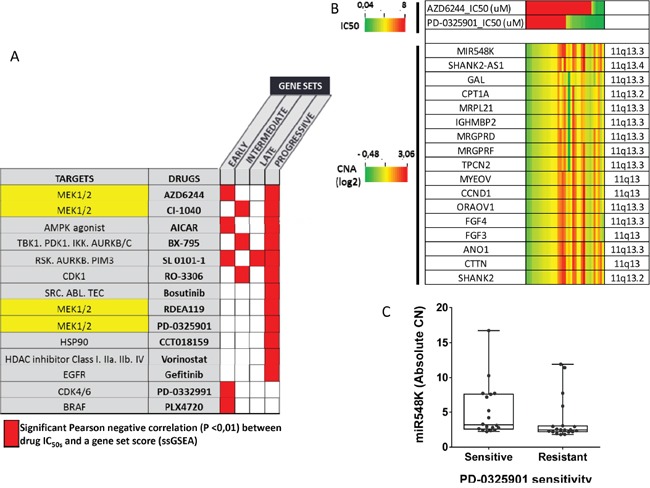
Correlation of enrichment scores for early (EGS), intermediate (IGS), late (LGS) and progressive (PGS) gene subsets with *in vitro* sensitivity to 98 drugs in established squamous cell carcinoma cell lines from the head and neck, esophagus and lung **A.** Enrichment scores for EGS, IGS, LGS and PGS were computed in 22 head and neck, 23 esophageal SCC and 6 SCC of the lung and correlated with the half maximal inhibitory concentration (IC_50_) of 98 drugs, downloaded from the GDSC [24]. Most significant negative correlations are shown in red (*P*<0.01). Main drug target(s) are shown. **B.** Using data from CCLE [28], we show the association of Copy Number (log2 values) in 11q13 and sensitivity to 2 MEK inhibitors (AZD6244, PD-0325901), in 37 cell lines (5 head and neck, 15 esophageal and 17 SCC of the lung). **C.** Then, we compared miR548K absolute copy number between sensitive cell lines to PD-0325901 (IC50 <8μM) and resistant cell lines (IC50>8μM). Copy number in miR548 K (region 11q13) is higher in sensitive cell lines (Mann Whitney test, *P*=0.0421)

We then investigated whether cell lines enriched for the 4-NQO derived gene sets were associated with specific patterns of drug sensitivity. For this, we used gene expression and drug sensitivity data downloaded from “The Genomics of Drug Sensitivity in Cancer” (GDSC) project [24, 25]. We selected cell lines established from SCC of the head and neck, esophagus and lung because they are included in the same cancerization field associated with cigarette smoking and they share common genomic alterations (Supplementary Table 8). EGS, IGS, LGS and PGS enrichment scores were computed in a total of 51 SCC cell lines and were correlated with the IC_50_ of 98 drugs. With a cut-off *P*-value of less than 0.01, we identified 14 drugs negatively correlated with at least one of the 4-NQO-derived gene sets. Most of them were inhibitors of the MAPK/ERK pathway and of the cell cycle (Figure 4A). The PGS ES was correlated with sensitivity to a variety of drugs. Those included an mTOR inhibitor (AMPK agonist) and an EGFR inhibitor (gefitinib), two targets that have been previously reported to prevent oral cancer development in the 4-NQO model [26, 27]. Interestingly, all four MEK1/2 inhibitors included in the panel of 98 drugs were significantly correlated with the ES for EGS, IGS and PGS subsets (Figure 4A). Consistent with the results obtained in the TCGA OSCC dataset (Figure 3A), and using drug sensitivity and CNA data from CCLE [28], we observed a trend for association of copy number gain in 11q13.3 with increased sensitivity to the two MEK1/2 inhibitors (PD-0325901 and AZD6244) (Figure 4B) tested in 37 head and neck, esophagus and lung cell lines (Supplementary Table 8). Considering cell lines with IC50 below 8μM to be sensitive to PD-0325901 and cell lines with IC50 above 8μM to be resistant to PD-0325901, we found higher miR548K copy number in sensitive cell lines (*P*=0.0421) (Figure 4C).

### The 4-NQO derived gene sets in human OPL and their association with outcome

**Figure 5:**
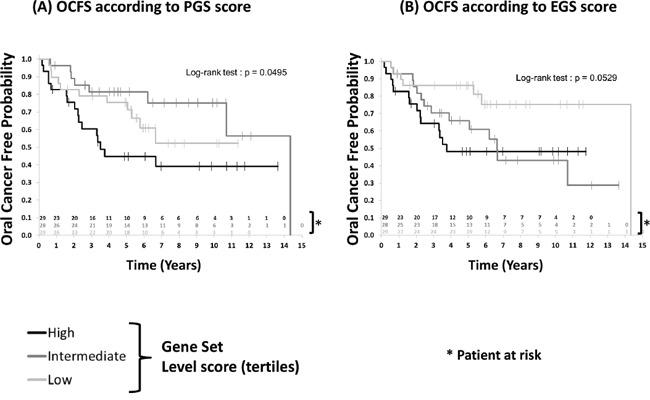
Patients with oral preneoplasia enriched for the mouse early (EGS) or progressive (PGS) gene sets and oral cancer free survival (OCFS) Enrichment scores were computed as detailed in in 86 patients with oral preneoplasia included in a chemoprevention trial (GSE26549) [11] (see Material and Methods section). Higher enrichment scores for EGS (*P*=0.0529) and PGS (*P*=0.0495) were associated with poor oral cancer-free survival. Statistical significance was given by the log-rank test.

Finally, we tested whether patients with OPL (oral premalignant lesions) enriched for 4-NQO derived gene subsets were at higher risk of developing OSCC. Gene expression profiles of 86 OPL collected in a prospective cohort of 86 patients were used (GSE26549) [11]. In this study, 35 patients developed an OSCC as the median follow-up was 7 years (95% CI [5.6-8.6]). Enrichment scores of these 86 OPL were computed for TGS, EGS, IGS, LGS, and PGS. For each gene subset, 3 groups of patients were defined by dividing the ES into tertiles (low, intermediate, high score). The Kaplan Meier analysis revealed a tendency of association between a poor OCFS and higher ES for the EGS (Log-rank test p=0.0529) and PGS (Log-rank test p= 0.0495) (Figure 5). No significant association was found between OCFS and TGS, IGS and LGS (Supplementary Figure 3). (page 8)

Although univariate Cox analyses suggested a prognostic effect on OCFS of EGS ES (HR High vs Low =2.997 [1.149-7.816]; HR Intermediate vs Low=2.64 [1.013-6.878], p=0.0681) and PGS ES (HR High vs Low =1.711 [0.790-3.707]; HR Intermediate vs Low=0.604 [0.233-1.562], p=0.0681) respectively, multivariate analyses failed to confirm these results once adjusted on age and histology (Supplementary Table 9).

### Overview of the results obtained across datasets

**Figure 6:**
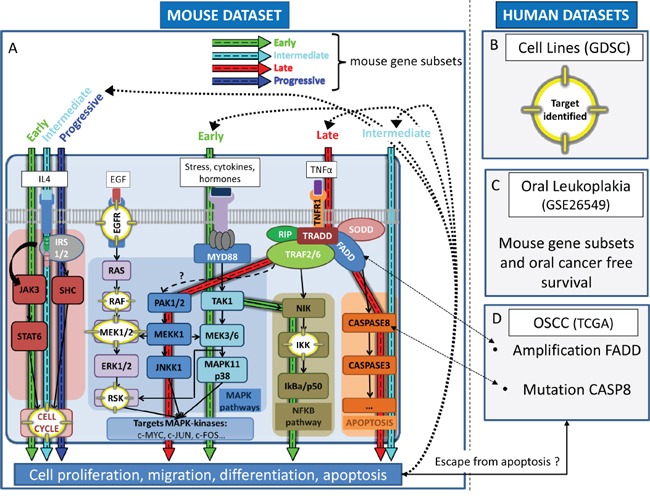
Overview of the dynamic expression changes observed in the 4-NQO model projected in pathways pertinent to human oral preneoplasia (OPL) and squamous cell carcinoma (OSCC) This figure shows the coherence between main molecular events occurring in the 4-NQO mouse model (EGS, IGS, LGS, PGS) and those involved in human oral preneoplasia and OSCC. Mitogen-activated protein kinase (MAPK), apoptosis, cell cycle, and the nuclear factor NF-κB (NFKB) pathways are dysregulated during oral tumorigenesis and were found to be correlated (p<0.01) with one or more of mouse gene subsets as indicated by blue (PGS), green (EGS), red (LGS) and turquoise (IGS) arrows respectively (A). Those correlations were consistent with drug sensitivity patterns in established squamous cell carcinoma cell lines; corresponding targets are highlighted (yellow) within the pathways **A, B.** Patients whom OPL were enriched for the mouse EGS were at increased risk of developing OSCC (borderline statistical significance) **C.** OSCC enriched for the mouse TGS harbor genomic features (amplification region 11q13) that may be associated with increased sensitivity to MEK1/2 inhibitors **D.** Abbreviations: TCGA: The Cancer Genome Atlas; GDSC: Genomics of Drug Sensitivity in Cancer; EGS: Early Gene Subset; IGS: Intermediate Gene Subset; LGS: Late Gene Subset; PGS: Progressive Gene Subset.

In the 4-NQO model, enrichment of the NFKB pathway was observed at early stages, and the activation of MAPK/ERK signaling pathway, was enriched at both early (significant enrichment of the NTHI pathway) and late (significant enrichment of the TNFR1 pathway) stages (Figure 6A). Consistently, we found that *in vitro* (Figure 6B), sensitivity to inhibitors of the MAPK/ERK or NFKB pathways (IKK) were correlated to the ES of 4-NQO-derived gene subsets. Similarly, IL4 pathway, that involves activation of the cell cycle, was found to be enriched in the 4-NQO model, consistent with the efficacy of CDK4/6, CDK1, and AURKB/C inhibitors cell cycle inhibitors *in vitro*. A trend toward association of EGS and PGS with a poor OCFS is in favour of the clinical relevance of association of EGS and PGS with these biological pathways (Figure 6C). Finally, three pathways involving apoptosis were found to be enriched in the 4-NQO model, which was consistent with frequent *FADD* CN gains and *CASP8* mutations in OSCC enriched in TGS (Figure 6D).

## DISCUSSION

By analyzing the dynamics of gene expression changes in the 4-NQO model of oral tumorigenesis and by projecting those changes in various human *in vitro* and *in vivo* datasets, we have shown that the 4-NQO is a relevant mouse model for human OSCC with frequent *CASP8* mutations and amplifications at 11q13.3, that may be sensitive to MEK inhibition. This work is in line with a recent observation of cross-species genomic conservation between preclinical models of HNSCC and HNSCC in humans [29]. We also illustrate the value of studying preclinical models of early tumorigenesis to help defining new strategies for prevention and therapy of HNSCC.

*CASP8* mutations have been reported in 8-10% HNSCC [30–32]. They are considered as either loss-of-function through suppression of apoptosis initiated by this apoptosis related-gene [30], or gain-of-function mutations conferring resistance to death receptor, or enhancing migration, invasion and tumor growth *in vitro* [32]. *CASP8* has been associated with *HRAS* mutations [31] and seems to define a subgroup of HPV-negative oral cancers recently identified by the presence of activating mutations in *HRAS*, inactivating *CAPS8* mutations and wild type *TP53* [33]. In our analysis, TCGA tumors enriched for the 4-NQO TGS were less frequently mutated for *PIK3CA*; which goes in line with the fact that *PIK3CA* mutations are found in 6-11% HNSCC, mostly in HPV-positive and especially in pharyngeal cancers [33–35]. These observations suggest that *CASP8* mutations may be involved in smoking-related tumorigenesis of the oral cavity, while *PI3KCA* may be more frequently involved in OSCC developing in the population of never smokers. The 4-NQO model may be pertinent to smoking-associated OSCC, but is likely not relevant to OSCC affecting non-smokers or HPV-associated oropharyngeal SCC.

Amplification in 11q13.3 was another genomic feature associated with OSCC enriched for the 4-NQO derived TGS. This region includes *MIR548K*, a microRNA recently characterized as a novel oncogene in esophageal squamous cell carcinomas (ESCC), a tumor entity that shares common risk factors and genomic alterations with HNSCC [36]. Depletion of *MIR548K* has been shown to suppress cellular growth and mobility, although its targets have not been reported. Targeting *MIR548K* may be relevant in the context of chemoprevention of tumors included in the same cancerization field.

The association between the enrichment mouse-derived gene subsets in OPLs validates the relevance of the 4-NQO model for the study of new chemoprevention agents. We observed a trend in association of the EGS and OCFS, suggesting that the key molecular events occur early during oral tumorigenesis. This observation is consistent with our previous report of a strong association between gene expression changes in OPL and the risk of developing OSCC, beyond clinical and pathological variables, including histological changes [11].

Beyond risk assessment, we seek to identify new candidate drugs for chemoprevention. The concept of field cancerization refers to the effects of chronic exposure to smoking and alcohol in patients with or without cancer, with progressive onset of molecular alterations in initially histologically and clinically normal epithelia [37]. The observation of the authors was that the grossly normal epithelium adjacent to the invasive carcinoma frequently demonstrated dysplasia or carcinoma *in situ*. Later, the concept of field cancerization was supported by the identification of similar genetic alterations in matched dysplastic and malignant lesions from the oral cavity [4]. The use of molecular markers has validated the concept of field cancerization. Within the context of the recently proposed paradigm termed “reverse migration”, we tested the association between the enrichment of SCC cell lines for mouse-derived gene sets with drug sensitivity in order to identify new candidate drugs that may also benefit patients in the context of prevention [38, 39]. We took advantage of the resources available from the GDSC project. We selected SCC cell lines from various anatomical sites, including the head and neck, esophagus and lung because they were all established from tumors included in the cancerization field. Most significant correlations were observed with inhibitors of the MAPK/ERK pathway and of the cell cycle. This was consistent with the results of the GSEA analysis in mouse showing a significant enrichment of gene sets involved in cell cycle and proliferation in agreement with a recent Gene Ontology analysis in the 4-NQO model [40]. The MAPK/ERK pathway includes many targetable kinases [41]. Interestingly, the ES for the PGS was associated with sensitivity to number of drugs, particularly inhibitors of the MAPK pathway including AZD6244 (MEK1/2), CI-1040 (MEK1/2), SL0101-1 (RSK, AURKB, PIM3), RDEA119 (MEK1/ 2), PD-0325901 (MEK1/2) and gefitinib (EGFR) targeting members of this signaling cascade. Some may affect predominantly the MAPK/ERK pathway while others affect the MAPK/JNK pathway, confirming that MAPK in an important node in OSCC. Although difficult in the prevention setting, combination therapies may help addressing the challenge of acquired resistance to EGFR inhibition for patients with HNSCC [42]. These observations suggest that MEK1/2 inhibition may be of interest to prevent OPL malignant transformation or to treat OSCC harboring *CASP8* mutation and/or an amplification at 11q13.3. Of note, our study was based on the analysis of a small number of mouse samples that may have hindered other significant pathways involved during oral tumorigenesis.

In conclusion, the study of the dynamics of molecular changes in the 4-NQO mouse model is relevant to identify new strategies for both the prevention of oral cancer development and the treatment of OSCC. In particular, MEK1/2 inhibition may be of interest to prevent the development of OSCC in patients with OPL or to treat patients with OSCC harboring a *CASP8* mutation and/or amplification at 11q13.3.

## MATERIALS AND METHODS

### Mouse specimens

The animal protocol was approved by the University of Texas M.D. Anderson Cancer Center Animal Care and Use Committee. A total of 52 male CBA mice were included in the study. 4-NQO was administered in half of them in their drinking water at the dose of 100 μg/mL for 8 weeks as described elsewhere [14]. Drink water was changed every week. Four mice treated with 4-NQO and four control mice were sacrificed every four weeks. Immediately following death, tongues were excised, longitudinally bisected, each half of the tongue was frozen on dry ice, embedded in OCT and stored at −80°C. By week 24, experiment was completed.

Sections of mouse tongues allowed to identify histologic changes associated with oral tumorigenesis including normal mucosa in untreated mice tongue, and hyperplasia, dysplasia, and tumor in 4-NQO treated mice tongue. Sections were 12 μm thick with a 6 μm section every 10 sections; all were stored at −80°C in order to preserve RNA quality. A half-tongue required about 100 slides with 2 sections per slide to be fully analyzed. Thinner sections were fixed in 70% ethanol and stained with hematoxylin & eosin (H&E) for a precise mapping of pathological changes by a veterinary pathologist (A.T.). Criteria used for histologic diagnoses were similar to those established in human [43]. Hyperplasia was defined by a thickened keratinized layer, with or without a thickened spinous layer (acanthosis), and an absence of nuclear or cellular atypia. Dysplasia was defined by enlarged nuclei and cells, large and/or prominent nucleoli, increased nuclear to cytoplasmic ratio, hyperchromatic nuclei, dyskeratosis, increased and/or abnormal mitotic figures, bulbous or teardrop-shaped rete ridges, loss of polarity, and loss of typical epithelial cell cohesiveness. As proposed by Hasina et al [14], the degree of dysplasia was not considered as it can be difficult even in human preneoplasia, with a high interobserver and intraobserver discordance rate [14, 44]. Biological triplicates of normal mucosa, hyperplasia, dysplasia and tumor from 3 different mice were selected for subsequent steps. Only typical pathological diagnoses were selected from mice that were sacrified at different time points, in order to avoid a potential bias due to the biological effect of age.

In order to limit our study to changes occurring in epithelial cells, we used an infrared laser microdissection system (Arcturus PixCell II®, Life Technologies, NY, USA) to isolate tongue epithelial cells from the underlying stroma and muscle. For microdissection, the 12-μm sections were stained with cresyl violet (Ambion® LCM staining kit, Life Technologies) that allows to preserve RNA quality. Microdissected cells on the cap were solubilized using a RLT lysis buffer and were subjected to total RNA extraction.

Total RNA extraction was carried out using the RNeasy Microkit® (Qiagen) and included an on-column DNase digestion. The RNA integrity number (RIN) was measured to evaluate the quality and quantity of each RNA sample, using the Agilent 2100 Bioanalyzer and RNA 6000 Pico Kit according to the manufacturer's instructions (Agilent Technologies, Santa Clara, CA, USA). Aliquots of RNA for genome-wide transcriptome analysis were prepared at the time of extraction. A total of 2 ng was used for RNA amplification using Ovation Pico WTA System v2 (NuGEN Technologies, San Carlos, CA, USA). Each amplified sense cDNA product was fragmented and biotinylated, using an Encore Biotin Module according to manufacturer's instructions (NuGEN Technologies). Each sample was hybridized onto a GeneChip Mouse Gene ST 2.0 Array (Affymetrix, Santa Clara, CA, USA) and imaged using the GeneChip Scanner 3000 7G (Affymetrix). Raw data were deposited in Gene Expression Omnibus (GEO) (GSE75421).

### Human datasets downloaded from public repositories for *in silico* analysis

Raw data of 3 independent datasets were downloaded from GEO. These included a set of 26 microdissected OSCC of the mobile tongue and 12 matching normal mucosa (GSE9844) [21]; a set of 167 OSCC, 17 unpaired oral dysplasia, and 45 oral cavity or oropharynx normal mucosa collected in patients who underwent a surgery for diseases other than cancer (GSE30784) [22]; and a set of 86 expression profiles from OPL biopsies collected prospectively in a chemoprevention trial, with a median follow-up of 7 years (95% CI [5.6-8.6]) and available outcome (GSE26549) [11]. A detailed description of these datasets is provided in Supplementary Table 7.

The Cancer Genome Atlas (TCGA) was queried [23] (last query in July 2014). Clinical and pathological information from 221 HPV negative OSCC and 24 matching normal oral mucosa were downloaded. Level 3 RNAseqv2 data were downloaded, corresponding to gene read counts that were generated from the IlluminaHiSeq platform and normalized using MapSplice [45] to do the alignment and RSEM [46] to perform the quantitation. Then a log2 transformation was performed. Mutation and copy number (CN) data were downloaded from cBioPortal [47]. Among genes found to be significantly mutated by MutSig, we selected those mutated in at least 5% of HNSCC. The putative copy number (−2, −1, 0, 1 or 2) and the log_2_ CN given by GISTIC for 310 genes with recurrent CN alterations (CNAs) in HNSCC were downloaded. Combined mutation and CN data was available for 152 patients with OSCC.

Gene expression raw data for 51 cell lines as well as drug sensitivity data (half maximal inhibitory concentration, IC_50_) for 144 drugs tested in those cell lines were downloaded as well from “The Genomics of Drug Sensitivity in Cancer” (GDSC) project [24, 25]. Combined expression profiles and drug sensitivity data were available for 98 drugs in 51 squamous cell carcinoma cell lines from the head and neck (n=22), the esophagus (n=23) and the lung (n=6). We also used drug sensitivity and copy number alteration data of 37 cell lines from Cancer Cell Line Encyplopedia [28]. Description of cell lines is provided in Supplementary Table 8.

### Bioinformatics analysis

Data analysis was performed using the Array Studio software (Omicsoft Corporation) and the Bioconductor packages in the R language (http://www.bioconductor.org) [48]. Raw data from microarrays were processed using quantile normalization and the robust multi-array average (RMA) algorithm and were log_2_ transformed [49]. Hierarchical cluster analysis using the Pearson correlation and Ward linkage method and principal component analysis (PCA) using the first 2 components were performed to provide an overview of the data and visualize the distribution of various samples according to the similarities between their expression profiles. Both analyses used either genome-wide expression profiles or specific gene sets as defined above.

Gene Set Enrichment Analysis (GSEA) was performed using the “pre-ranked” tool [19]. GSEA is a robust computational method that determines whether an *a priori* defined set of genes shows statistically significant differences between 2 biological states (in our case tumor vs. normal). GSEA aims to interpret large-scale expression data by identifying pathways and process. The input data for GSEA procedure were the following: i-a complete table of genes ranked according to the log_2_ transformed FC between tumor and normal mucosa, ii-a mapping file for identifying transcripts in the corresponding platform (GeneChip Mouse Gene ST 2.0 Array), and iii-a catalogue of functional gene sets from Molecular signature Database (MSigDB database v4.0 updated May 31, 2013, GSEA/MSigDB web site v4.02 released Jan 18, 2014). A total of 4722 curated gene sets (canonical pathway, chemical and genetic perturbations, BioCarta, GenMAPP, and KEGG gene sets) and 189 oncogenic signatures were available. Default parameters were used. Inclusion gene set size was set between 15 and 500 and the phenotype was permutated 1,000 times.

The single-sample GSEA (ssGSEA) projection tool from GenePattern [50] was used to compute separate enrichment scores (ES) for each sample of a given dataset using defined gene sets *i.e.* TGS, IGS, LGS, PGS subsets and BioCarta pathways from the Molecular Signature Database (MSigDB database v4.0 updated May 31, 2013; v4.02 released Jan 18, 2014, ref 20). The gene expression values for a given sample are rank-normalized, and an ES is produced using the empirical cumulative distribution functions of the genes in the gene set and the remaining genes [19, 20]. When UP and DN versions of a gene set are available, a combined score was computed. A rate of ES was computed for early (EGS), intermediate (IGS), late (LGS) and progressive (PGS) gene sets at each step of oral tumorigenesis: from “normal to hyperplasia” (H->N), from “hyperplasia to dysplasia” (D->H), and from “dysplasia to tumor” (T->D), using the following formula:

$$\frac{\left|\Delta \text{Gene set score}({\text{Step}}_{n}-{\text{Step}}_{n-1})\right|}{\left|\Delta \text{Gene set score}({\text{Tumor-N}}^{\text{al}})\right|}$$

### Statistical analysis

Unpaired two-sided Student's t-test or non-parametrical Mann-Whitney test were performed to compare continuous data between two groups and one-way ANOVA or Kruskal-Wallis test if more than 2 groups. Pearson's χ2 test or Fisher's exact test were used to analyze qualitative data. The Pearson Correlation Coefficient (r) was also estimated to measure the strength of a linear association between two continuous variables. The r coefficient can take values from +1 (positive association) to −1 (negative association). A value of 0 indicates no association.

About the analysis of the 86 samples from GSE26549 dataset, the median follow-up was estimated with the inversed Kaplan Meier method. Oral Cancer Free Survival (OCFS) distributions were estimated using the Kaplan-Meier method and compared with the Log-Rank test between groups of patients defined by tertiles of gene set enrichment scores (low, intermediate and high score). OCFS was defined as time from first biopsy to oral cancer or to the date of last follow-up (for censored patients). Univariate and multivariate Cox proportional hazard model, including age and histology, were built to investigate the potential association between the ES for TGS, EGS, IGS, LGS and PGS and oral cancer-free survival. All statistical tests were two-sided, and P-values <= 0.05 were considered to be statistically significant.

The statistical analysis was performed using GraphPad Prism version 6.00 (San Diego, SA) and SAS software version 9.3 (SAS Institute Inc, Cary, NC, USA).

## SUPPLEMENTARY FIGURES AND TABLES












