# Supplementary material for: The dynamics of gene expression changes in a mouse model of oral tumorigenesis may help refine prevention and treatment strategies in patients with oral cancer
Source: Oncotarget. 2016 Mar 24;7(24):35932–45. doi: 10.18632/oncotarget.8321 (PMC5094973; doi:10.18632/oncotarget.8321)
Supplement: Supplementary file 1 [file oncotarget-07-35932-s001.pdf]

The dynamics of gene expression changes in a mouse model of oral tumorigenesis may help refine prevention and treatment strategies in patients with oral cancer

SUPPLEMENTARY FIGURES AND TABLES

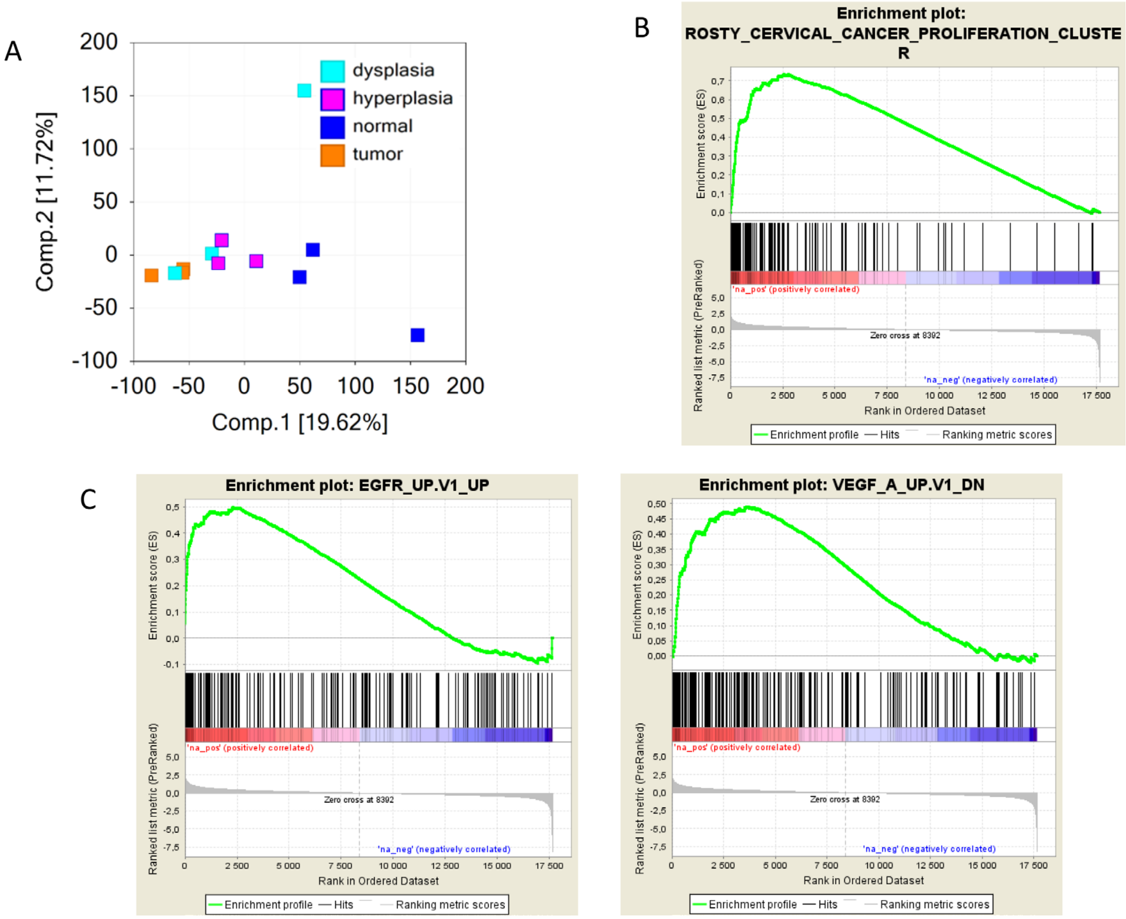

Supplementary Figure S1: Gene expression changes in tumor vs normal samples in the 4-NQO model. A. Principal Component Analysis of the 12 samples using TGS. **B-C.** Gene set enrichment analysis (GSEA) using the fold-change between tumor and normal samples as the input (see Material and Methods section). We show the top gene sets positively enriched among the 4722 curated gene sets (B) and the 186 oncogenic signatures (C) available from Molecular signature Database (19).

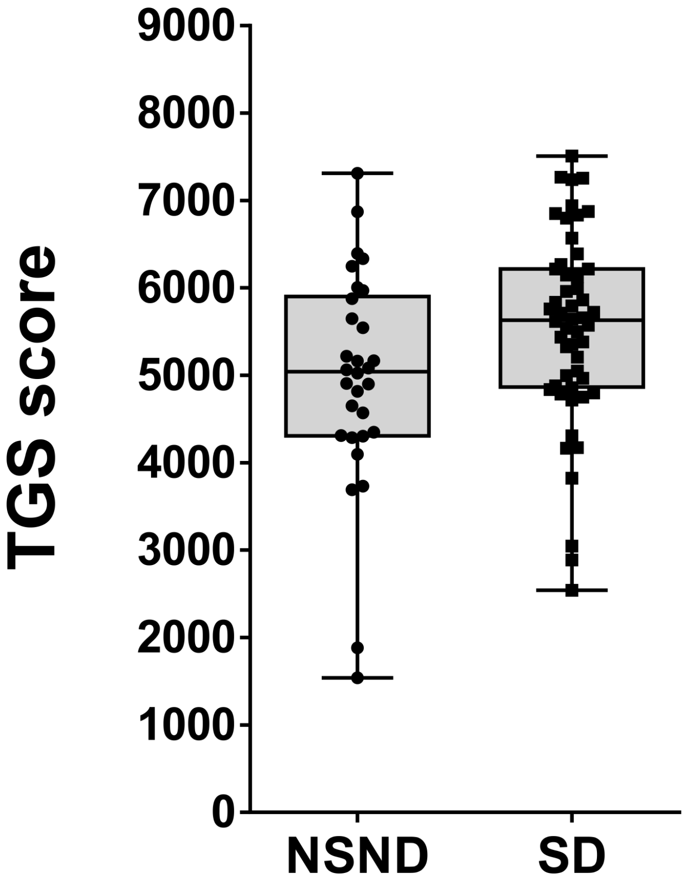

Supplementary Figure S2: The 4-NQO model is relevant to transcriptome changes observed in human disease during oral carcinogenesis. Enrichment scores (ES) were computed for the TGS in the TCGA set of 221 OSCC including 30 never-smokers & never-drinkers (NSND) and 52 current smokers and drinkers (SD). ES of NSND and SD were compared using a Student's test. We found that SD had a higher ES than NSND (*P*=0.0400).

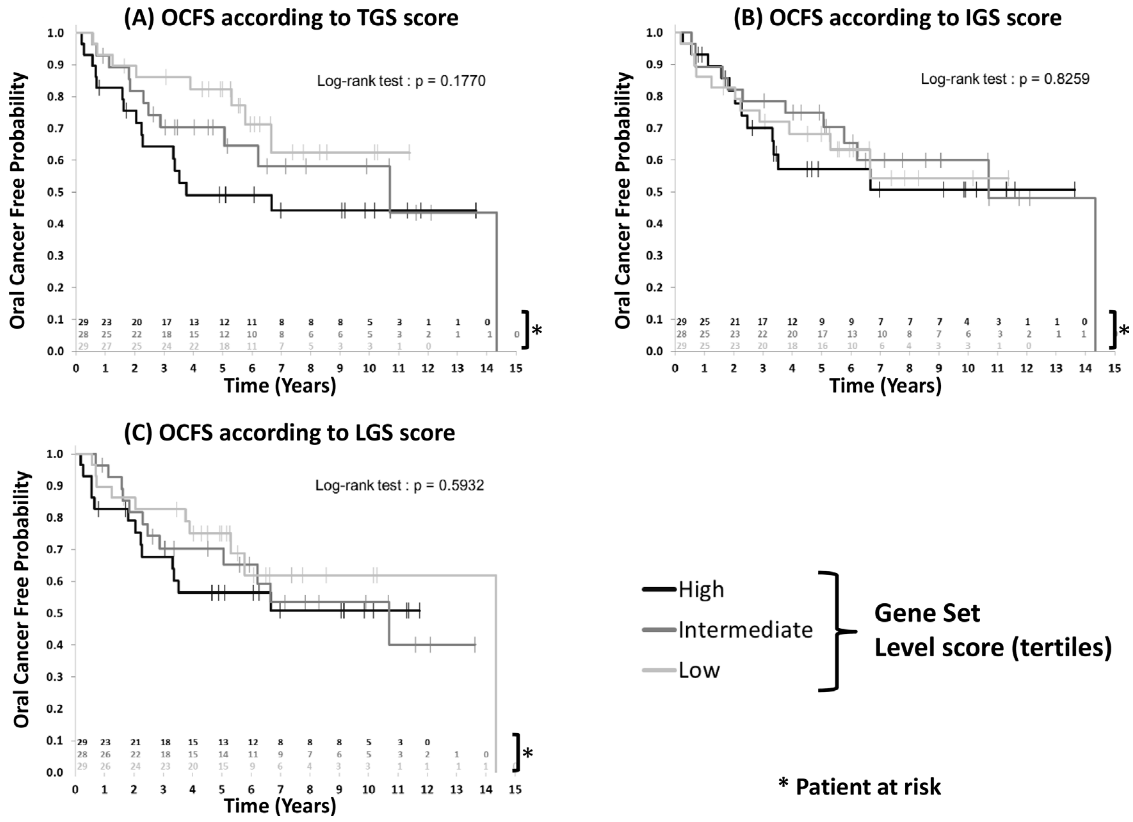

Supplementary Figure S3: Association of ES for IGS LGS and TGS, with oral cancer free survival. Enrichment scores were computed as detailed in in 86 patients with oral preneoplasia included in a chemoprevention trial (GSE26549) (see Material and Methods section). High enrichment scores for TGS (*P*=0.1770), IGS (*P*=0.8259) and LGS (*P*=0.5932) were not associated with oral cancer-free survival. Statistical significance was given by the log-rank test. Abbreviations: IGS: Intermediate Gene Subset; LGS: Late Gene Subset; TGS: Tumor Gene Set.

Supplementary Table S1: Gene expression profiles of the 4-NQO mouse model of oral tumorigenesis: technical details and samples description.

See Supplementary File 1

Supplementary Table S2: Number of mouse and corresponding human orthologous genes identified in the tumor gene set (TGS) and non-overlapping early, intermediate, late and progressive gene subsets.

GENE SET	MOUSE		HUMAN	
Version	UP	DN	UP	DN
**Tumor**	584	753	514	508
**Early**	180	283	152	225
**Intermediate**	24	71	18	55
**Late**	57	50	49	35
**Progressive**	180	224	162	179

UP: overexpressed genes **DN**: underexpressed genes

Supplementary Table S3: Criteria used to define the "early", "intermediate", "late" and "progressive" gene subsets in the 4 NQO-model. Abbreviations: UP: transcripts up-regulated; DN: transcripts downregulated; T: tumor; H: hyperplasia; Nal: normal; D: dysplasia; FC: fold-change

		T vs. Nal	H vs. Nal	D vs. H	T vs. D
**Early**	UP	log2FC >1	log2FC > 1	---	---
	DN	log2FC < -1	log2FC <-1	---	---
**Intermediate**	UP	log2FC >1	-1 <log2FC < 1	log2FC > 1	---
	DN	log2FC < -1	-1 <log2FC < 1	log2FC <-1	---
**Late**	UP	log2FC >1	-1 <log2FC < 1	-1 <log2FC < 1	log2FC > 1
	DN	log2FC < -1	-1 <log2FC < 1	-1 <log2FC < 1	log2FC <-1
**Progressive**	UP	log2FC >1	0 ≤ log2FC ≤ 1	0 ≤ log2FC ≤ 1	0≤ log2FC ≤ 1
	DN	log2FC < -1	-1 < log2FC < 0	-1 < log2FC < 0	-1 ≤ log2FC <0

Supplementary Table S4: Details of mouse gene sets and their corresponding human orthologous genes.

See Supplementary File 2

Supplementary Table S5: Details of Biocarta pathways.

See Supplementary File 3

Supplementary Table S6: The biological significance of mouse-derived gene sets was assessed by correlating their enrichment score with the enrichment score for 208 Biocarta pathways. Pearson correlations were computed. Abbreviations: EGS: Early Gene Subset; IGS: Intermediate Gene Subset; LGS: Late Gene Subset; PGS: Progressive Gene Subset.

See Supplementary File 4

Supplementary Table S7: Studies used to validate gene sets identified in the 4-NQO mouse model in human datasets

GEO series	Sample type	N	Platform	Ref
**GSE9844**	**Oral samples**	**38**	Affymetrix Human Genome U133 Plus 2.0 Array	(1)
	Oral tongue SCC	26		
	Normal mucosa	12		
**GSE30784**	**Oral samples**	**229**	Affymetrix Human Genome U133 Plus 2.0 Array	(2)
	Oral SCC	167		
	Dysplasia	17		
	Normal mucosa	45		
**GSE26549**	**Oral leukoplakia**	**86**	Affymetrix Human Gene 1.0 ST Array	(3)
**E-MTAB-783 (GDSC)**	**Cancer Cell Lines**	**51**	Affymetrix Human Genome U133A Array	(4,5)
	Head and Neck	23		
	Esophagus	22		
	Lung	6		
**GSE36133 (CCLE)**	**Cancer Cell Lines**	**51**	Affymetrix Human Genome U133 Plus 2.0 Array	(6)
	Head and Neck	23		
	Esophagus	22		
	Lung	6		

1. Ye H, Yu T, Temam S, Ziober BL, Wang J, Schwartz JL, et al. Transcriptomic dissection of tongue squamous cell carcinoma. BMC Genomics. 2008;9:69.
2. Chen C, Méndez E, Houck J, Fan W, Lohavanichbutr P, Doody D, et al. Gene expression profiling identifies genes predictive of oral squamous cell carcinoma. Cancer Epidemiol Biomark Prev Publ Am Assoc Cancer Res Cosponsored Am Soc Prev Oncol. août 2008;17:2152-62.
3. Saintigny P, Zhang L, Fan Y-H, El-Naggar AK, Papadimitrakopoulou VA, Feng L, et al. Gene expression profiling predicts the development of oral cancer. Cancer Prev Res Phila Pa. févr 2011;4:218-29.
4. Garnett MJ, Edelman EJ, Heidorn SJ, Greenman CD, Dastur A, Lau KW, et al. Systematic identification of genomic markers of drug sensitivity in cancer cells. Nature. 29 mars 2012;483:570-5.
5. Yang W, Soares J, Greninger P, Edelman EJ, Lightfoot H, Forbes S, et al. Genomics of Drug Sensitivity in Cancer (GDSC): a resource for therapeutic biomarker discovery in cancer cells. Nucleic Acids Res. janv 2013;41:D955-61.
6. Barretina J, Caponigro G, Stransky N, Venkatesan K, Margolin AA, Kim S, et al. The Cancer Cell Line Encyclopedia enables predictive modelling of anticancer drug sensitivity. Nature. 29 mars 2012;483:603-7.

Supplementary Table S8: Description of the cell lines used for *in silico* analysis. Gene expression and drug sensitivity (IC_{50}) data were downloaded from "The Genomics of Drug Sensitivity in Cancer" (GDSC) project (n=51) and the Cancer Cell Line Encyclopedia (n=37).

See Supplementary File 5

Supplementary Table S9: Univariate (A) and multivariate (B) Cox proportional hazard model for oral cancer-free survival, including age, histology, and the enrichment score (either the early, intermediate, late, progressive gene subsets or the tumor gene set).

(A)

Variable	N	HR	IC 95%	p-value
**Tumor Gene Set**	86			0.1897
Low		1	-	
High		2.217	[0.936-5.251]	
Intermediate		1.549	[0.621-3.862]	
**Early Gene Subset**	86			0.0681
Low		1	-	
High		2.997	[1.149-7.816]	
Intermediate		2.64	[1.013-6.878]	
**Intermediate Gene Subset**	86			0.8269
Low		1	-	
High		1.162	[0.511-2.642]	
Intermediate		0.899	[0.388-2.082]	
**Late Gene Subset**	86			0.5979
Low		1	-	
High		1.55	[0.660-3.642]	
Intermediate		1.361	[0.571-3.244]	
**Progressive Gene Subset**	86			0.0603
Low		1	-	
High		1.711	[0.790-3.707]	
Intermediate		0.604	[0.233-1.562]	

(B)

Variable		HR	IC 95%	p- value
**Tumor Gene Set**				
**Score**	Low	1	---	0.5436
	High	1.663	[0.673-4.113]	
	Intermediate	1.342	[0.533-3.380]	
**Age**	For one supplementary year	1.025	[0.994-1.057]	0.1104
**Histology**	Dysplasia	1	---	0.2139
	Hyperplasia	0.637	[0.313-1.297]	
**Early Gene Subset**				
**Score**	Low	1	---	0.1839
	High	2.468	[0.920-6.622]	
	Intermediate	2.187	[0.821-5.821]	
**Age**	For one supplementary year	1.024	[0.994-1.055]	0.1242
**Histology**	Dysplasia	1	---	0.2919
	Hyperplasia	0.685	[0.338-1.385]	
**Intermediate Gene Subset**				
**Score**	Low	1	---	0.535
	High	0.853	[0.364-1.998]	
	Intermediate	0.616	[0.256-1.479]	
**Age**	For one supplementary year	1.034	[1.003-1.065]	0.0324
**Histology**	Dysplasia	1	---	0.0933
	Hyperplasia	0.556	[0.280-1.104]	
**Late Gene Subset**				
**Score**	Low	1	---	0.816
	High	1.323	[0.539-3.249]	
	Intermediate	1.259	[0.521-3.040]	
**Age**	For one supplementary year	1.029	[1-1.059]	0.0532
**Histology**	Dysplasia	1	---	0.1968
	Hyperplasia	0.622	[0.302-1.279]	
**Progressive Gene Subset**				
**Score**	Low	1	---	0.1865
	High	1.222	[0.527-2.832]	
	Intermediate	0.536	[0.201-1.381]	
**Age**	For one supplementary year	1.024	[0.993-1.057]	0.1269
**Histology**	Dysplasia	1	---	0.1608
	Hyperplasia	0.598	[0.292-1.227]	
