# Supplementary material for: The dynamics of gene expression changes in a mouse model of oral tumorigenesis may help refine prevention and treatment strategies in patients with oral cancer
Source: Oncotarget. 2016 Mar 24;7(24):35932–45. doi: 10.18632/oncotarget.8321 (PMC5094973; doi:10.18632/oncotarget.8321)
Supplement: Supplementary file 6 [file oncotarget-07-35932-s006.docx]

**Supplementary Table 8: Description of the cell lines used for *in silico* analysis**. Gene expression and drug sensitivity (IC50) data were downloaded from “The Genomics of Drug Sensitivity in Cancer” (GDSC) project (n=51) and the Cancer Cell Line Encyclopedia (n=37).

| CELL LINE | DATA BASE | Histology | Tissue |
| --- | --- | --- | --- |
| CAL27 | CCLE & SANGER | squamous cell carcinoma | head_and_neck |
| FADU | CCLE & SANGER | squamous cell carcinoma | head_and_neck |
| HSC2 | CCLE & SANGER | squamous cell carcinoma | head_and_neck |
| KYSE140 | CCLE & SANGER | squamous cell carcinoma | oesophagus |
| KYSE150 | CCLE & SANGER | squamous cell carcinoma | oesophagus |
| KYSE180 | CCLE & SANGER | squamous cell carcinoma | oesophagus |
| KYSE410 | CCLE & SANGER | squamous cell carcinoma | oesophagus |
| KYSE450 | CCLE & SANGER | squamous cell carcinoma | oesophagus |
| KYSE510 | CCLE & SANGER | squamous cell carcinoma | oesophagus |
| KYSE520 | CCLE & SANGER | squamous cell carcinoma | oesophagus |
| KYSE70 | CCLE & SANGER | squamous cell carcinoma | oesophagus |
| NCIH2170 | CCLE & SANGER | squamous cell carcinoma | lung |
| NCIH226 | CCLE & SANGER | squamous cell carcinoma | lung |
| NCIH520 | CCLE & SANGER | squamous cell carcinoma | lung |
| SCC25 | CCLE & SANGER | squamous cell carcinoma | head_and_neck |
| SCC9 | CCLE & SANGER | squamous cell carcinoma | head_and_neck |
| SKMES1 | CCLE & SANGER | squamous cell carcinoma | lung |
| TE1 | CCLE & SANGER | squamous cell carcinoma | oesophagus |
| TE11 | CCLE & SANGER | squamous cell carcinoma | oesophagus |
| TE15 | CCLE & SANGER | squamous cell carcinoma | oesophagus |
| TE5 | CCLE & SANGER | squamous cell carcinoma | oesophagus |
| TE9 | CCLE & SANGER | squamous cell carcinoma | oesophagus |
| BB30HNC | SANGER | squamous cell carcinoma | head_and_neck |
| BB49HNC | SANGER | squamous cell carcinoma | head_and_neck |
| CAL33 | SANGER | squamous cell carcinoma | head_and_neck |
| Ca922 | SANGER | squamous cell carcinoma | head_and_neck |
| HN | SANGER | squamous cell carcinoma | head_and_neck |
| KYSE270 | SANGER | squamous cell carcinoma | oesophagus |
| KOSC2 | SANGER | squamous cell carcinoma | head_and_neck |
| LB771HNC | SANGER | squamous cell carcinoma | head_and_neck |
| A253 | SANGER | squamous cell carcinoma | head_and_neck |
| RPMI2650 | SANGER | squamous cell carcinoma | head_and_neck |
| SAS | SANGER | squamous cell carcinoma | head_and_neck |
| LK2 | SANGER | squamous cell carcinoma | lung |
| OE33 | SANGER | squamous cell carcinoma | oesophagus |
| DOK | SANGER | squamous cell carcinoma | head_and_neck |
| HO1N1 | SANGER | squamous cell carcinoma | head_and_neck |
| OE19 | SANGER | squamous cell carcinoma | oesophagus |
| HSC3 | SANGER | squamous cell carcinoma | head_and_neck |
| HCE4 | SANGER | squamous cell carcinoma | oesophagus |
| COLO680N | SANGER | squamous cell carcinoma | oesophagus |
| Detroit562 | SANGER | squamous cell carcinoma | head_and_neck |
| ECGI10 | SANGER | squamous cell carcinoma | oesophagus |
| TE10 | SANGER | squamous cell carcinoma | oesophagus |
| BHY | SANGER | squamous cell carcinoma | head_and_neck |
| EPLC272H | SANGER | squamous cell carcinoma | lung |
| SCC4 | SANGER | squamous cell carcinoma | head_and_neck |
| TE6 | SANGER | squamous cell carcinoma | oesophagus |
| TE8 | SANGER | squamous cell carcinoma | oesophagus |
| SCC15 | SANGER | squamous cell carcinoma | head_and_neck |
| TE12 | SANGER | squamous cell carcinoma | oesophagus |
| Calu1 | CCLE | squamous cell carcinoma | lung |
| EBC1 | CCLE | squamous cell carcinoma | lung |
| HARA | CCLE | squamous cell carcinoma | lung |
| HCC15 | CCLE | squamous cell carcinoma | lung |
| KNS62 | CCLE | squamous cell carcinoma | lung |
| KYSE30 | CCLE | squamous cell carcinoma | oesophagus |
| LC1sqSF | CCLE | squamous cell carcinoma | lung |
| LOUNH91 | CCLE | squamous cell carcinoma | lung |
| LUDLU1 | CCLE | squamous cell carcinoma | lung |
| NCIH1869 | CCLE | squamous cell carcinoma | lung |
| OE21 | CCLE | squamous cell carcinoma | oesophagus |
| RERFLCAI | CCLE | squamous cell carcinoma | lung |
| Sq1 | CCLE | squamous cell carcinoma | lung |
| SW1573 | CCLE | squamous cell carcinoma | lung |
| SW900 | CCLE | squamous cell carcinoma | lung |
